# Genistein Supplementation Affects Mineral Homeostasis in Rats with Mammary Cancer

**DOI:** 10.3390/foods15061040

**Published:** 2026-03-16

**Authors:** Dorota Skrajnowska, Arkadiusz Szterk, Karol Ofiara, Paweł Kowalczyk, Bartosz Strus, Barbara Bobrowska-Korczak

**Affiliations:** 1 Department of Toxicology and Food Science, Faculty of Pharmacy, Medical University of Warsaw, Banacha 1, 02-097 Warsaw, Poland; dorota.skrajnowska@wum.edu.pl (D.S.); s078007@student.wum.edu.pl (P.K.); 2 ASLAB Science, Fort Służew 1/9, 02-787 Warsaw, Poland; a.szterk@aslabsci.com (A.S.); k.ofiara@aslabsci.com (K.O.); b.strus@aslabssci.com (B.S.); 3 Department of Pharmacology and Toxicology, Warsaw University of Life Sciences, Nowoursynowska 166, 02-787 Warsaw, Poland

**Keywords:** genistein, calcium, magnesium, potassium and sodium distribution, mineral homeostasis, cancer

## Abstract

**Background:** The aim of our study was to analyze the supply of various forms of genistein (nano, micro, and classic) on the content of four macroelements—calcium, magnesium potassium, and sodium—in the kidneys, brains, hearts, livers, spleens and femurs of rats under conditions of mammary gland neoplasia (induced by 7,12-dimethylbenz[a]anthracene (DMBA)). **Methods**: Thirty-two 30-day-old Sprague-Dawley rats were included in this study. The animals were randomly assigned to four experimental groups: the control group received only a standard diet (without supplementation), while three groups were supplemented with genistein in different forms—nanoparticles (0.1 mg/mL; size 92 ± 41 nm), microparticles (0.1 mg/mL; size 587 ± 83 nm), or macromolecular genistein (0.1 mg/mL). To induce mammary gland cancer, all rats were administered DMBA. **Results**: In the presented studies, significant changes in the content of elements in the organs of rats supplemented with various forms of genistein were observed. Of particular importance was the occurrence of soft tissue calcifications caused by the dietary supplementation of rats with various forms of genistein, ranging from the classic form to the nanometric form, in the context of an existing mammary gland neoplastic process. Calcium accumulation occurred in various tissues—the brain (from 252% to 449%); the heart (from 159% to 661%); the liver (from 90% to 613%), regardless of the form of genistein; and the spleen (by 127%) and femurs (by 294%) only in the case of nanogenistein supplementation—compared to rats from the control group not supplemented with any form of genistein in conditions of induced mammary gland cancer. **Conclusions**: Genistein supplementation in cancer conditions affects mineral homeostasis in rats.

## 1. Introduction

Genistein is a lipophilic compound with a low molecular weight, which makes it easily absorbed but poorly soluble in water. This is one of the reasons for its nonlinear pharmacokinetics, and increasing the dose alone cannot improve its bioavailability (saturation of metabolic enzymes) [1,2]. ADME studies have shown that although genistein has beneficial absorption properties in the intestines, its poor solubility may prevent the absorption of higher doses without appropriate formulations [1,3]. Miniaturization associated with the development of nanotechnology can effectively increase bioavailability and also create the possibility of the targeted delivery of, for example, genistein to a specific tissue, thereby enhancing its therapeutic potential [4,5,6]. However, changing the size of a given molecule to the nanoscale modifies its properties, mainly due to the increased surface-to-volume ratio and frequent aggregation of nanoparticles, which further alters the biological response [7]. Thus, it is risky to assess the safety of nanocomposites based on the toxicological and ecotoxicological profile of the substance on a normal scale, especially since the distribution of classic genistein affects almost all organs and tissues. In a study by Coldham and Sauer [8], the distribution of genistein in rats was assessed after the oral administration of 14C-labeled genistein, and radioactivity was detected in every organ. The highest levels of radioactivity were found in the gastrointestinal tract, liver, and kidneys. Intermediate concentrations were found in the testes and ovaries, uterus, prostate, and vagina, while low concentrations of radioactivity were found in the brain, thymus, spleen, skeletal muscles, bones, and adipose tissue. Zhou et al. [2] observed the highest concentrations of genistein (after oral administration) in the gastrointestinal tract and in the livers and kidneys of rats. The concentrations in the reproductive organs were equal to those in skeletal muscle and adipose tissue. The authors emphasize that at high doses, the absorption, biotransformation, and excretion of genistein were nonlinear and dose-dependent.

The results of the cited studies indicate the presence of genistein throughout the body, raising questions about the therapeutic effects of consuming large amounts of genistein, genistein analogs, or soy-based nutraceuticals and nanoforms. Research on the distribution of genistein is closely related to its effect on individual organs. In the case of nanoformulations, it would be best to determine the biological assessment of nanoparticles taking into account particle size, organ/tissue, and physiological status (health/disease). In general, the classic form of genistein is considered a safe, well-studied supplement ingredient and can be used in very high doses without side effects. The anticancer and LDL-lowering effects of genistein are observed at doses of 10–20 mg/kg body weight per day [9]. In contrast, the recommended dose for osteoporosis therapy is 40 mg to 110 mg/day [10]. Available information also indicates that the ADME of isoflavones differs between rodents and humans, so oral bioavailability values in rodents should be carefully extrapolated to humans. Human studies provide some information on oral bioavailability based on the percentage of the dose recovered in urine. However, the concentration of isoflavones in urine is poorly correlated with the maximum serum concentrations, indicating the limitations of urine measurements as a predictor of systemic bioavailability [11].

The cited literature shows that genistein, when administered orally, is distributed throughout all organs. Their functioning can be modified via various mechanisms, especially in the context of coexisting neoplastic processes. It is worth emphasizing the therapeutic potential of genistein’s anticancer activity by blocking tyrosine kinase receptors, which can affect various growth factors and the formation of new blood vessels that nourish cancer cells and reduce the risk of cancer metastasis [12,13,14].

Exposure to isoflavones, in a well-studied Asian population or from clinical trials (for doses of 100 mg/day), does not adversely affect the risk of breast cancer or the thyroid endocrine system in healthy women. However, for risk groups, such as people with iodine deficiency, subclinical hypothyroidism, or congenital or non-congenital thyroid dysfunction, as well as women with breast cancer or a family history of breast cancer, it is recommended not to take isoflavone supplements and to limit the amount in the diet to 50 mg/day [11].

In our previous studies, we found very large changes in the content of 14 elements, especially calcium, in the femurs and kidneys of rats after dietary supplementation with nanogenistein [15,16]. In this article, we studied additional rat organs to determine whether calcium migration and that of three other macroelements (Mg, Na, and K) will also affect them. It turned out that calcium deposition also affects the heart, brain, liver, and spleen of rats (in the latter case, only for nanogenistein supply). This study evaluated the impact of three forms of genistein on mineral elements in the organs of breast cancer-induced rats. The findings clarified the potential side effects of nanogenistein in cancer models. This research provides a reference for the clinical application of genistein in oncology patients.

## 2. Materials and Methods

The miniaturized form of nanogenistein (micro- and nanoform) was obtained by conventional grinding and homogenization. The preparation of genistein (micro- and nanoparticles) and parameters for evaluating the average particle size and zeta potential of the particles have been described in our previous work [17].

### 2.1. Laboratory Animals

This study used female Sprague-Dawley rats (*n* = 32, 30 days) from the Animal Laboratory, Department of General and Experimental Pathology, Medical University of Warsaw. The work protocol was approved by Ethics Committee (code 645/2018).

The animals received water and Labofeed H feed (standard diet: Labofeed H, Żurawia 19, 89-240 Kcynia, Poland; composition: 22.0% protein, 4.0% fat, 30.0% starch, 5.0% fiber, 6.5% minerals) ad libitum. They were kept in a room with a temperature of 22 °C with a 12 h light–dark cycle. The observation period was planned for 100 days. After a 10-day adaptation period to the experimental conditions, the animals were randomly divided into 4 groups of 8 individuals. The first group—the control group (*n* = 8)—received only standard feed, without any supplementation, while the second group, in addition to the standard diet, received a water suspension of classic genistein, hereinafter referred to as macrogenistein (*n* = 8), in a dose of 0.4 mL at a concentration of 0.1 mg/mL, i.e., 0.2 mg/kg bw; the third group received a water suspension of miniaturized genistein (587 ± 83 nm), hereinafter referred to as microgenistein (*n* = 8) in an amount of 0.4 mL at a dose of 0.1 mg/mL, i.e., 0.2 mg/kg bw; the fourth group received an aqueous suspension of miniaturized genistein (92 ± 41 nm), hereinafter referred to as nanogenistein (*n* = 8), in a volume of 0.4 mL at a dose of 0.1 mg/mL, i.e., 0.2 mg/kg bw. To induce mammary gland cancer, all rats were administered DMBA (7,12-dimethylbenz[a]anthracene (DMBA: Sigma-Aldrich, St. Louis, MO, USA)) in rapeseed oil twice, on day 60 of life at a dose of 80 mg/kg body weight and on day 80 of life at a dose of 40 mg/kg body weight.

Interactive factors were eliminated by applying the same experimental procedure to all rats, i.e., age, experimental time, feed, housing conditions, tumor induction method, and supplementation method. The control group (standard diet—without supplementation) was given 0.4 mL of water instead of genistein to induce a similar level of stress to the animals in the control group.

The animals were examined by palpation during this study to characterize the time course of tumor development. All data related to the time of appearance and number of cancerous tumors caused by chemical induction (7,12-dimethylbenz[a]anthracene) are described in an earlier publication [17].

### 2.2. Determination of Trace Elements

The reagents and solvents used in the analysis were of the highest commercially available purity. The water used to prepare the standard solutions and test samples was obtained from the Barnstead NANOpure Diamond UV system (resistance 18 MΩ cm^−1^). Additionally, 65% HNO_3_ and 37% HCl, Suprapur (Merck, Darmstadt, Germany), were used to digest the samples. The standard solutions of the elements Ca, Mg, Na and K had concentrations of 1000 mg/L (from Merck (Germany)). The plasma gas (argon) and the gas in the collision chamber (helium) had a purity of over 99.999%.

### 2.3. Sampling

On the 100th day of this study, the kidneys, brains, hearts, livers, spleens, and femurs of the rats were collected and frozen at −80 °C. Before the actual analysis, the samples were thawed, and then tissue mineralization was performed by dissolving them in a mixture of acids (1 mL of HCL and 4 mL of HNO_3_) and mineralizing them in hermetically sealed Teflon vessels. Samples were digested in a high-pressure laboratory microwave. The heating program was carried out in two steps using different temperatures and pressures depending on the type of tissue. Following digestion, the samples were diluted with water to a final volume of 100 mL.

### 2.4. Instrumental Analysis

For kidneys, hearts, brains, livers, and spleens, the level of calcium was analyzed using a 7800 quadrupole ICP-MS (Agilent Technologies, Minato City, Tokyo, Japan) containing an octopole collision chamber.

For the determination of elements such as K, Mg, and Na, the analysis was performed using flame atomic absorption spectrometers—Solar GF Zeeman and iCE3500 (Thermo Fisher Scientific 168 Third Avenue, Waltham, MA, USA)—containing single cavity cathode lamps, using an air/acetylene flame. The wavelengths for the determination of K, Mg, and Na were 285.2, 766.5, and 589.0 nm, respectively. The analytical methods used were verified using certified material (water matrix reference material: EnviroMAT sewage, high (EU-H), catalog number 140-025-138, batch number S160225019 from SCP Scienc, Baie-D’Urfé, QC, Canada).

### 2.5. Statistical Analysis

Statistica 12.0 software (StatSoft, Tulsa, OK, USA) was used for statistical analysis. The normal distribution of the data was tested using the Shapiro–Wilk method. For the normal data, Student’s *t*-test and an ANOVA, followed by Tukey’s test, were used for analysis. The non-normal data was analyzed with a Mann–Whitney U nonparametric test. The results were considered statistically significant when *p* ≤ 0.05.

## 3. Results

**Figure 1:**
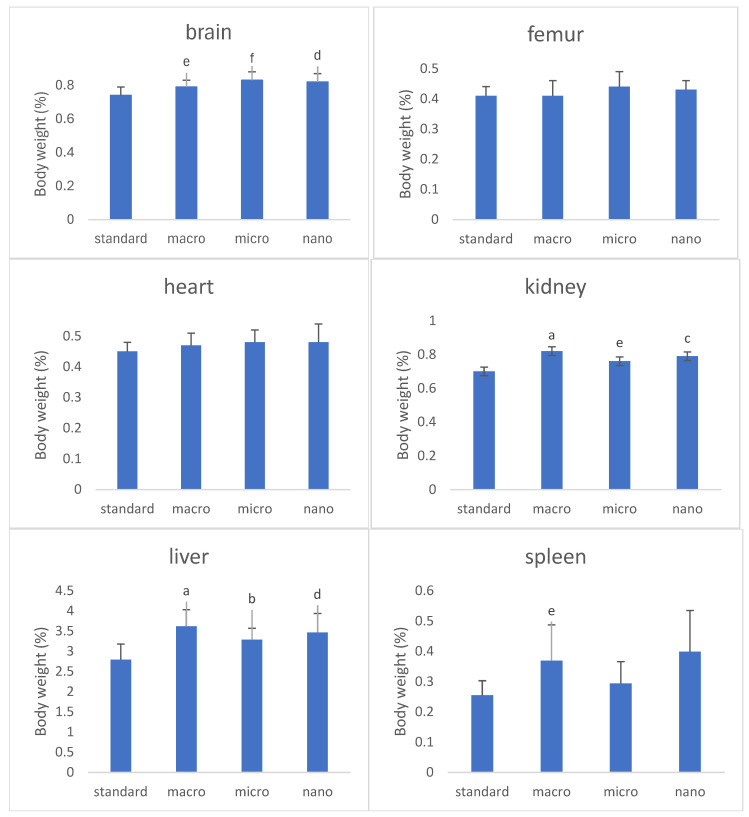
The weights of kidneys, femurs, brains, livers, spleens, and hearts relative to the final body weight of rats (%) g/100g. %—The ratio of bone/organ weight to body weight was expressed in grams per 100 g of body weight. Statistically significant results: a—*p* ≤ 0.001; b—*p* ≤ 0.02; c—*p* ≤ 0.01; d—*p* ≤ 0.01; e—*p* ≤ 0.05; f—*p* ≤ 0.005 compared to the standard group.

The results of organ weights in rats supplemented with various forms of genistein, namely macrogenistein, microgenistein, and nanogenistein, and those without supplementation (standard group) in relation to final body weight are shown in Figure 1 and Table S1.

This experiment did not reveal any differences in final body weight, femur weight, heart weight, or spleen weight (except for a 44% increase in the macrogenistein group).

Changes were observed in kidney weight in the groups supplemented with macrogenistein, microgenistein, and nanogenistein (increases of 17%, 9%, and 12%, respectively) and in liver weight (increases of 29%, 18%, and 24%, respectively) compared to the standard group (Figure 1, Table S1).

A significant increase was also observed in brain weight in all supplemented groups. Brain weight increased by 7–12% depending on the group compared to the standard group (Figure 1, Table S1).

**Figure 2:**
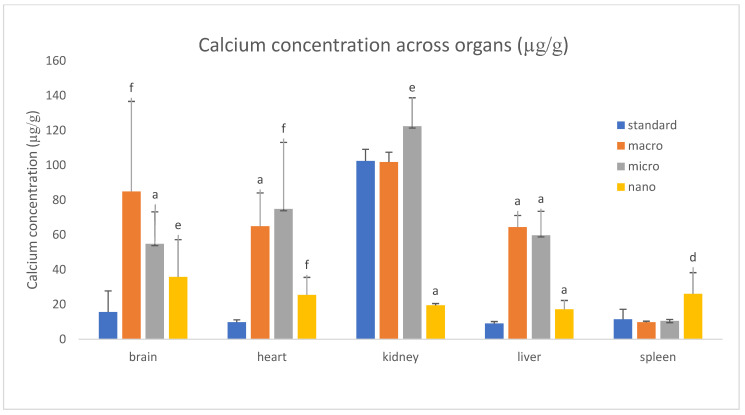
Content of calcium in rats’ organs (μg/g) (*n* = 32). Results are presented as means ± SEM. a—*p* ≤ 0.001; d—*p* ≤ 0.01; e—*p* ≤ 0.05; f—*p* ≤ 0.005 compared to standard group.

**Figure 3:**
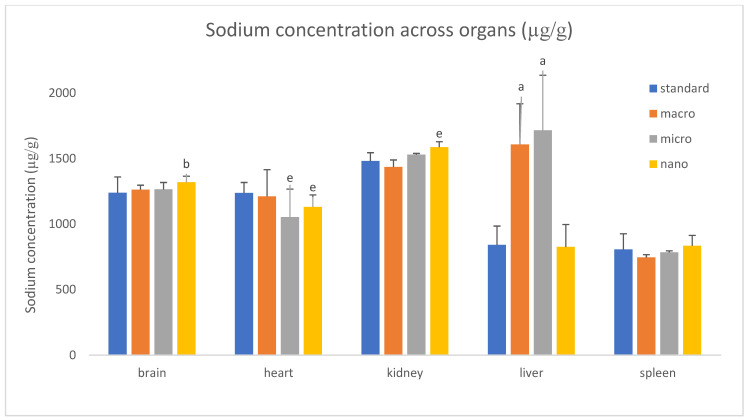
Content of sodium in rats’ organs (μg/g) (*n* = 32). Results are presented as means ± SEM. a—*p* ≤ 0.001; b—*p* ≤ 0.02; e—*p* ≤ 0.05 compared to standard group.

**Figure 4:**
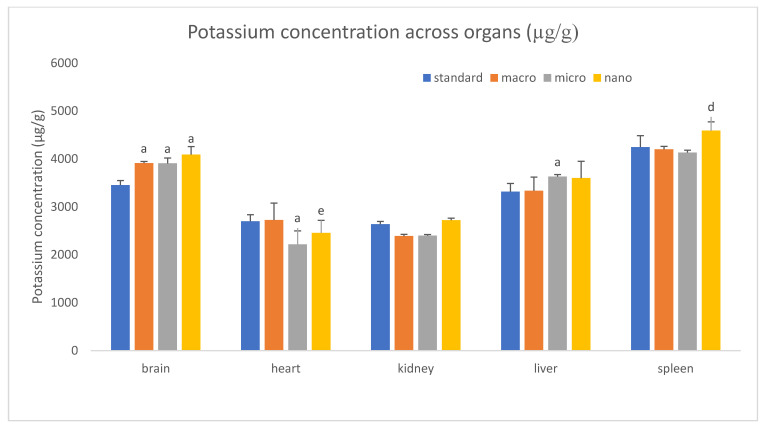
Content of potassium in rats’ organs (μg/g) (*n* = 32). Results are presented as means ± SEM. a—*p* ≤ 0.001; d—*p* ≤ 0.01; e—*p* ≤ 0.05 compared to standard group.

**Figure 5:**
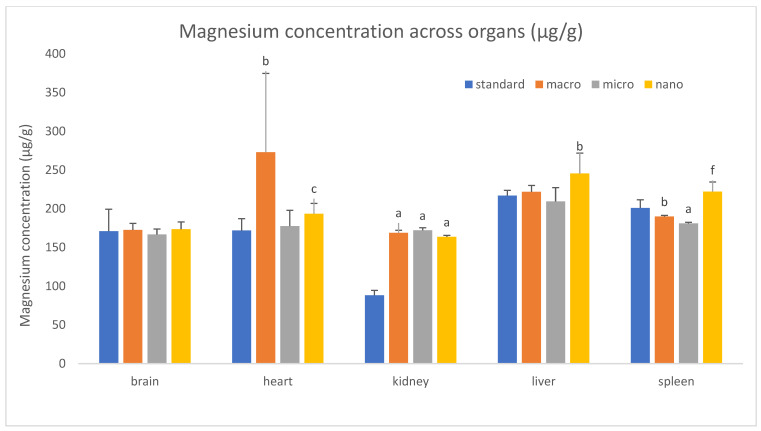
Content of magnesium in rats’ organs (μg/g) (*n* = 32). Results are presented as means ± SEM. a—*p* ≤ 0.001; b—*p* ≤ 0.02; c—*p* ≤ 0.01; f—*p* ≤ 0.005 compared to standard group.

**Figure 6:**
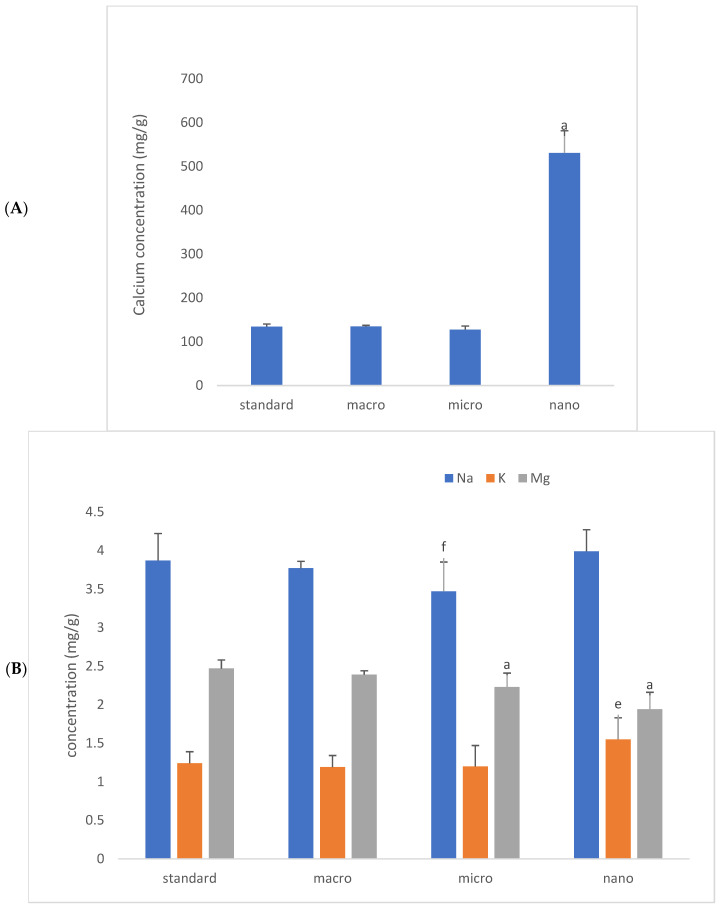
Content of calcium (mg/g) (**A**), sodium, potassium and magnesium (mg/g) (**B**) in rats’ femurs (mg/g) (*n* = 32). Results are presented as means ± SEM. a—*p* ≤ 0.001; e—*p* ≤ 0.05; f—*p* ≤ 0.005 compared to standard group.

**Figure 7:**
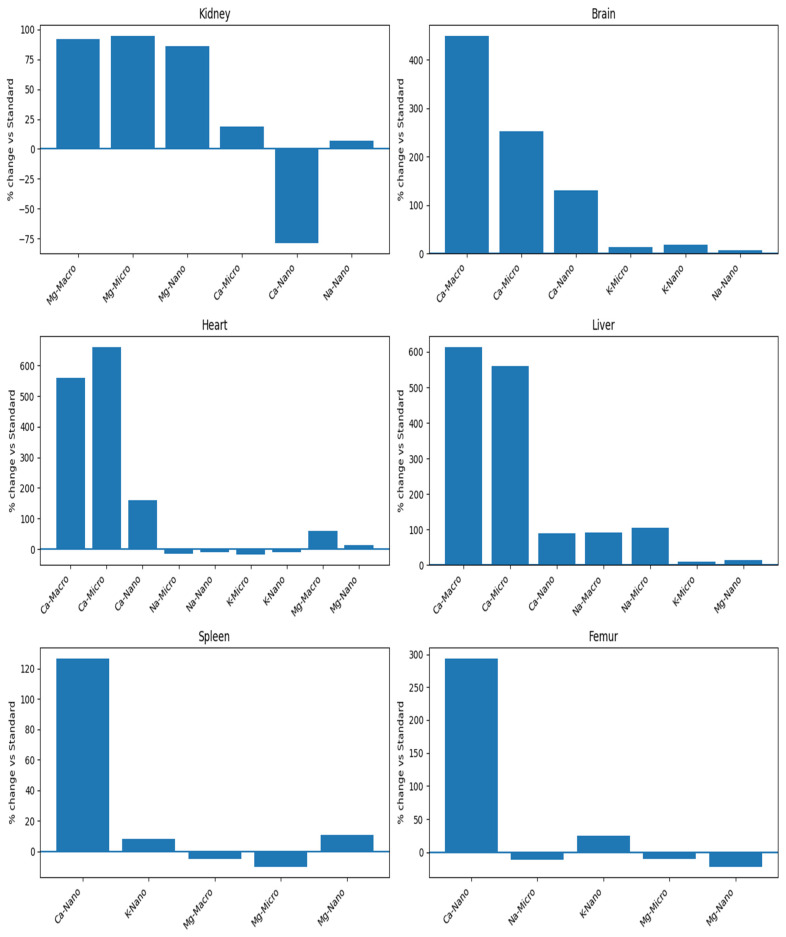
Summary of percentage changes in concentration of elements in organs and tissues.

We analyzed the basic mineral composition (Ca, Na, K, Mg) of organs in rats supplemented with various forms of genistein, namely macrogenistein, microgenistein, and nanogenistein, and those without supplementation (standard) (Figure 2, Figure 3, Figure 4, Figure 5, Figure 6 and Figure 7; Table S2).

The content of calcium and other macroelements changed significantly in virtually all organs examined (Figure 2, Figure 3, Figure 4, Figure 5, Figure 6 and Figure 7, Table S2). And so, the following observations were made:

Kidneys—In the group supplemented with nanogenistein, there was a 79% decrease in calcium levels, in contrast to the microgenistein group, where a 19% increase was observed compared to the unsupplemented group of rats. Regardless of the form of genistein used, strong increases in magnesium were observed, ranging from a minimum of 86% (nanogenistein) to a maximum of 95% (microgenistein).

Brain—Regardless of the form of genistein used, strong increases in calcium were recorded, ranging from a minimum of 130% (nanogenistein) to a maximum of 449% (macrogenistein). There was also a several percent increase in potassium levels in the micro- and nanogenistein groups compared to the standard group. A 7% increase in sodium content was also noted in the nanogenistein-supplemented group compared to the standard group.

Heart—Regardless of the form of genistein used, strong increases in calcium were observed, ranging from a minimum of 159% (nanogenistein) to a maximum of 661% (microgenistein). There was also a decrease in potassium and sodium levels in the microgenistein group (15% and 18%, respectively) and nanogenistein group (9% each) compared to the standard group. There was also an increase in magnesium levels of 59% in the macrogenistein group and 13% in the nanogenistein group compared to the standard group.

Liver—Regardless of the form of genistein used, strong increases in calcium were noted, ranging from a minimum of 90% (nanogenistein) to a maximum of 613% (macrogenistein). There was also a 9% increase in potassium levels in the microgenistein group and magnesium (13%) in the nanogenistein group. An increase in sodium content was also observed—by approximately 100% in the macrogenistein and microgenistein groups compared to the standard group.

Spleen—In the nanogenistein-supplemented group, there was an increase in calcium, potassium, and magnesium by 127%, 8%, and 11%, respectively, and a decrease in magnesium by 5% and 10% in the macro- and microgenistein groups compared to the standard group.

Bones—In the nanogenistein-supplemented group, there was an increase in calcium levels of 294% and potassium levels of 25% and a decrease in magnesium levels of 22% compared to the standard group. There was also a 10–11% decrease in sodium and magnesium content in the group of rats receiving microgenistein compared to the non-supplemented group.

## 4. Discussion

This study evaluated the impact of three forms of genistein on mineral elements in the organs of rats under conditions of chemically induced mammary gland neoplasia. The time of tumor appearance, their number, and size were described in detail in our previous work [17,18]. The incidence of cancer was 100% in the groups without supplementation (grade II adenoma) and in the groups with macrogenistein (grade II adenoma) and nanogenistein (grade III adenoma) and 88% in the group with microgenistein (grade III adenoma) (Tables S3 and S4) [17,18]. However, the rate of tumor development increased by one week in the microgenistein group and by two weeks in the nanogenistein group compared to animals receiving only a standard diet. In the presented studies, significant changes in the contents of elements in the organs of rats supplemented with various forms of genistein were observed. Of particular importance was the occurrence of soft tissue calcifications caused by the dietary supplementation of rats with various forms of genistein, ranging from the classic form to the nanometric form, in the context of an existing mammary gland neoplastic process. The question remains open as to whether changes in the concentrations of elements in the examined organs, especially calcium, can be attributed to the effects of cancer, the effects of genistein, or the synergism of both factors.

Calcium is an essential mineral, 99% of which is found in bones and teeth. Sometimes calcium deposits accumulate in soft tissues, resulting in tissue hardening. Calcium deposits can cause problems with blood vessels (atherosclerosis) and individual organs such as the kidneys, lungs, brain, pancreas, joints, tendons, and mammary glands [3,19,20,21]. Calcification most often occurs in damaged or dead tissue. It can be mild or intense and sometimes malignant. Over time, bone tissue, which is the main store of calcium, naturally degenerates, and this can cause the released calcium salts to accumulate in other tissues. However, the calcification process can also be stimulated by factors unrelated to age. If calcification occurs in healthy tissue, it is closely related to excess calcium in the blood, sometimes associated with excess vitamin D, kidney problems or hyperthyroidism, diet (e.g., containing phytoestrogens) or cancer, or other factors. Soft tissue calcifications are common and are divided into four categories according to the mechanism of formation, dystrophic, iatrogenic, metastatic, and idiopathic, depending on clinical and biochemical correlations.

It is known that the work of the heart and kidneys is closely related and that a disorder in one of them can cause dysfunction in the other, leading to cardiorenal syndrome [22,23]. Both organs work together to regulate vascular tone, diuresis, blood pressure, natriuresis, intravascular volume homeostasis, and peripheral tissue perfusion. Renal excretion plays a major role in maintaining electrolyte balance in the body, so changes in renal function can affect electrolyte concentrations in the heart. Potassium, magnesium, sodium, and calcium play a key role in heart function [22]. Moving through the semipermeable cell membrane, they generate an action potential that triggers myocardial contraction and proper heart function. An imbalance of these electrolytes can contribute to arrhythmia and even cardiac arrest. Life-threatening arrhythmias are often associated with potassium disorders, especially hyperkalemia, and less commonly with disorders of serum calcium and magnesium concentrations [24]. In our study, Na and K in heart tissue decreased by several percent in the groups of rats supplemented with a modified form of genistein compared to the standard, unsupplemented group. This may indicate impaired conduction in the heart, probably due to excess calcium, as enormous calcification of the heart, amounting to several hundred percent, was found. Stimulated perhaps by the administration of genistein regardless of its form, strong increases in calcium in the heart (without significant changes in their mass)—from a minimum of 159% (nanogenistein) to a maximum of 560% (macrogenistein)—probably indicate the formation of calcium deposits in the coronary arteries. Coronary artery calcium (CAC) imaging is a specific marker of atherosclerosis with prognostic value in cardiovascular risk assessment [25]. Calcium affects myocardial cells through conduction, intracellular signaling, and muscle fiber contraction. Excessive calcium concentration can lead to a short QT interval (in electrocardiogram recordings), while calcium deficiency can cause a prolonged QT interval. In extreme cases, conduction disturbances can lead to cardiac arrest. Typical electrolyte disturbances include hyponatremia, hypokalemia, and hypomagnesemia [22].

It is worth noting that even the classic form of genistein administered to rats in such a small dose (0.2 mg/kg bw) in confirmed breast cancer caused an increase in calcium content in this tissue by as much as 560% compared to the group without genistein supplementation and also with diagnosed breast cancer.

The results obtained in our in vivo studies differ from the data presented in many publications emphasizing the beneficial role of genistein supplementation on the risk of developing cardiovascular diseases, which may be related to progressive cancer and/or impaired kidney function [16,26]. An increased magnesium content was also observed in the hearts of rats supplemented with classic genistein and, to a lesser extent, with nanogenistein modified to nano size, compared to the unsupplemented group. In the heart, magnesium plays a key role in modulating neuron excitation, impulse conduction, and myocardial contraction by regulating many ion transporters, including potassium and calcium channels [27]. Magnesium regulates proper vascular tone and prevents atherosclerosis, thrombosis, vascular calcification, and the proliferation and migration of endothelial and vascular smooth muscle cells. Since the kidneys are the main regulators of magnesium homeostasis, renal dysfunction can potentially lead to both magnesium depletion and overload, thereby increasing the risk of cardiovascular disease [27,28]. In our study, regardless of the form of genistein, there was an almost 100% increase in magnesium content in the kidneys of rats compared to the group on a standard unsupplemented diet. Magnesium ions inhibit phosphate-induced vascular calcification and impair calcium phosphate crystallization and, more specifically, the maturation of calprotectin particles. Given that excess phosphate causes kidney damage, magnesium may counteract this, as in the case of vascular calcification [28]. In any case, special attention should be paid to the use of genistein preparations in existing cancer, especially in hormone-dependent cancers. It appears that an imbalance of potassium, magnesium, sodium, and calcium can lead to serious cardiac complications.

Cancer affects not only the tissue directly attacked but the entire body, including the nervous system [29]. Neurological symptoms often affect cancer patients and may be associated with, among other things, disturbances in the homeostasis of individual elements in brain tissue. Patients diagnosed with malignant cancer seek the right diet that would support their primary therapy and at the same time improve their daily functioning, including reducing neurological symptoms that undoubtedly lower their quality of life.

Calcium and phosphorus are macronutrients that are primarily key building blocks of bones, but they also perform other tasks: calcium ions participate in intracellular communication, blood clotting, and muscle contractions, while phosphorus, as a component of compounds such as nucleic acids, proteins, and phospholipids, is involved in inheritance and cell signaling processes. Both calcium and phosphorus are essential for the proper functioning of brain tissue, and even slight changes in their concentration can have serious neurological consequences [30].

In our studies, we found a several percent increase in their mass in the brains of rats compared to the non-supplemented group and increases in calcium concentrations from 130% (nanogenistein) to a maximum of 449% (macrogenistein). There was also an increase of several percent in potassium levels in the micro- and nanogenistein groups compared to the standard group.

Computed tomography can detect intracranial calcifications within the brain parenchyma or vascular system [31]. There are many causes for this condition, ranging from age; dystrophy; or congenital, metabolic/endocrine or infectious defects to vascular, inflammatory, or neoplastic changes and toxic factors. It is believed that any disorder affecting calcium homeostasis can lead to calcifications in the brain, especially those caused by hypoparathyroidism, hyperparathyroidism, and hypothyroidism [32]. Hypercalcemia is a common problem among cancer patients [33]. Elevated serum calcium levels may also correlate with increased calcium concentrations in the brain [34]. There are no publications in the available literature on changes in calcium concentrations in the brain during chronic cancer. Furthermore, the observed increase in calcium concentration concerns the total calcium pool in the brain, without division into individual structures and calcium functions therein. An excessive intracellular calcium increase is associated with excitotoxicity and neurodegenerative processes. Calcium ions lead to the opening of a non-specific channel in the inner mitochondrial membrane and activate the superconducting system [35].

It is worth mentioning that neurological disorders occurring in cancer patients may result not only from the ongoing disease but also from an imbalance between elements in sensitive brain tissue, especially when using poorly controlled nanoparticle dietary supplements. Although nanoparticles are currently considered relatively safe, it is believed that excessive exposure may be associated with toxic effects. Brain tissue may prove to be a particularly sensitive organ, also due to its relatively easy crossing of the blood–brain barrier [36,37].

The next organ examined is the liver. Calcium content in the liver increased significantly in all groups supplemented with genistein from 90% to 613% compared to rats on a standard diet. The calcification of the liver can result from a number of causes: inflammation, infectious diseases, or vascular or proliferative diseases. The resulting scars, in turn, lead to the accumulation of small stones in the bile ducts of the liver. Over time, these stones become calcified, hard, and difficult to flush out. The liver gradually loses its detoxification efficiency and proper function. The liver is a multifunctional organ associated with the digestive tract and the metabolism of many compounds, including genistein [38]. The liver, being the main organ involved in the detoxification of xenobiotics, is particularly vulnerable to the effects of nanoparticles. Many studies have shown that nanoparticles, especially metallic ones, can accumulate in the liver and lead to adverse interactions with hepatocytes [39,40,41]. This is largely due to the fact that metallic nanoparticles are highly resistant to degradation, which can lead to their long-term persistence and, consequently, increasing toxicity and liver damage. It has been shown that long-term exposure to metallic nanoparticles induces inflammation as a result of oxidative stress, leading to, among other things, cancer, vascular disorders, fibrosis, and the loss of cellular functions such as xenobiotic detoxification.

Calcium is a versatile secondary messenger that regulates many liver functions, including lipid and carbohydrate metabolism, as well as bile secretion. Therefore, the dysregulation of calcium signaling is a characteristic feature of both acute and chronic liver diseases [42,43]. Furthermore, a growing body of evidence suggests that the combination of food quantity and quality, together with genetic susceptibility, can trigger the abnormal activation of innate immune signaling, which may contribute to chronic low-grade inflammation. The liver is a major player in the inflammatory response. Dietary/metabolic factors contribute to the pathogenesis of non-alcoholic fatty liver disease (NAFLD), the leading cause of liver disease in the Western world. The enlargement of the spleen, the central organ regulating the inflammatory immune response, is commonly observed in patients with NAFLD, representing the so-called “liver–spleen axis” [44].

The spleen is the largest and most underrated lymphatic organ in the human body. It plays a very important role in the body—it is responsible, among other things, for maintaining the balance of the lymphatic and circulatory systems. According to our research, calcium (by 127%) and magnesium (by 11%) levels increased significantly in the spleen only in the group receiving nanogenistein, in contrast to a slight decrease in magnesium concentration in the groups of rats supplemented with other forms of genistein.

It should be emphasized that changes in the spleen are relatively rare, and most of them are detected accidentally. Calcifications of the spleen can be single or multiple. The most common causes include residual granulomatous disease, sickle cell anemia, heart attacks, abscesses, cysts, inflammatory pseudomembranous tumors, hemangiomas, lymphomas, and metastases [45]. Splenic calcifications not associated with a mass, especially if they are numerous and punctate (starry sky pattern), do not require further diagnosis. Calcifications may occur in benign and malignant splenic masses. When a lesion shows thick-walled calcification (>5 mm), it can be diagnosed as benign [46,47]. Calcification may occur in non-lymphoma metastases, such as in the liver, especially secondary to neuroendocrine or colorectal tumors [46]. It has not yet been established whether splenic calcification may predispose patients to hyposplenism [48].

What could have caused such large, multi-organ changes in the content of macroelements, especially calcium, in the examined organs? There was undoubtedly a disturbance in mineral homeostasis, in particular calcium homeostasis, as a result of the action of genistein. Due to its chemical structure, this isoflavone acts as an agonist of the estrogen receptors ER-β, ER-α (estrogen receptor beta and alpha) and GPER (G protein-coupled estrogen receptor 1) [49,50]. Thus, genistein appears to be a powerful therapeutic agent as a substitute for hormone replacement therapy. Genistein is also used in experimental studies on the prevention of breast and prostate cancer or the inhibition of angiogenesis in cancerous tumors [51]. On the other hand, however, some reports indicate that genistein may also accelerate the proliferation of certain cancer cells or reduce their sensitivity to drugs currently used in chemotherapy [51]. The effect of genistein in the context of cancer therapy probably depends on the specific dose used. Can genistein affect calcium homeostasis? Probably yes. Many publications have demonstrated the beneficial effects of genistein and daidzein treatment in regulating calcium homeostasis and phosphate homeostasis and aspects of bone metabolism, thyroid function, and the pituitary–adrenal axis in a rat model of andropause, which appropriately reflects the disturbed physiological environment caused by the aging process [52,53].

Thus, the safe use of phytoestrogens in various diseases has been proven in many studies, but their use in oncology still requires consideration. Many researchers argue that the lack of uniformity between the recorded clinical data and the complexity of assessing the usefulness of most compounds make it impossible to reach a clear conclusion at this point [54,55]. And although genistein is the best-studied compound in this group, there is insufficient evidence of its selectivity to recommend it to a specific group of patients for the prevention of breast cancer, let alone to patients already suffering from breast cancer.

## 5. Conclusions

In summary, the presented studies show the massive calcification of organs and disturbances in the homeostasis of other elements in organs, especially in the case of nanogenistein administration. It confirms the need for a thorough toxicological analysis of new forms of active substances.

## Data Availability

The data presented in this study are available on request from the corresponding author.

## Supplementary Materials

The following supporting information can be downloaded at https://www.mdpi.com/article/10.3390/foods15061040/s1, Table S1: Weights of kidneys, femurs, brains, livers, spleens, and hearts relative to the final body weight of rats (%) g/100 g. Table S2: Content of basic minerals in rat organs. Table S3: Cancer induction in 7,12-dimethylbenz[a]anthracene treated rats in relation to supplementation [17,18]. Table S4: Histopathological examination of rats tumors [17,18].
