# Supplementary material for: Genistein Supplementation Affects Mineral Homeostasis in Rats with Mammary Cancer
Source: Foods. 2026 Mar 16;15(6):1040. doi: 10.3390/foods15061040 (PMC13025938; doi:10.3390/foods15061040)
Supplement: Supplementary file 1 [file foods-15-01040-s001.zip › foods-4177749-supplementary.pdf]

Table S1. Weights of kidneys, femurs, brains, livers, spleens, and hearts relative to the final body weight of rats (%) g/100g).

	Kidney (g)	%	Femur (g)	%	Brain (g)	%	Final body weight (g)
			Standard				
X	1.636	0.7	0.969	0.41	1.716	0.74	233.3
SD	0.197	0.06	0.110	0.03	0.143	0.05	17.3
			Macrogenistein				
X	1.750	**0.82**[a]	0.873	0.41	1.714	**0.79**[e]	214.5
SD	0.104	0.05	0.087	0.05	0.098	0.04	7.1
			Microgenisteina				
X	1.708	**0.76**[e]	0.993	0.44	1.873	**0.83**[f]	225.9
SD	0.082	0.04	0.057	0.05	0.068	0.05	13.9
			Nanogenistein				
X	1.745	**0,79**[c]	0.950	0.43	1.805	**0.82** [d]	221.1
SD	0.145	0.07	0.048	0.03	0.085	0.05	10.4

Diet	Liver (g)	%	Spleen (g)	%	Heart (g)	%	Final body weight (g)
Standard			Standard				
X	6.525	2.792	0.598	0.255	1.048	0.45	233,3
SD	1.097	0.388	0,135	0.048	0.121	0.03	17,3
			Macrogenistein				
Macrogenistein							
X	7.748	**3.618**[a]	0.793	**0.369**[e]	1.080	0.47	214,5
SD	0.759	0.411	0.263	0.118	0.111	0.04	7,1
			Microgenisteina				
Microgenistein							
X	7.415	**3.289**[b]	0.666	0.294	1.084	0.48	225,9
SD	0.629	0.283	0.184	0.072	0.091	0.04	13,9
			Nanogenistein				
Nanogenistein							
X	7.674	**3.466**[d]	0.753	0.399	1.058	0.48	221,1
SD	0.159	0.469	0.299	0.136	0.113	0.06	10,4

Results are presented as means ± SEM; statistically significant results are bolded

a - p≤0.001; b - p≤0.02; c - p≤0.01; d - p≤0.01; e - p≤0.05; f - p≤0.005 compared to the standard group

% - The ratio of organ weight to body weight of animals is expressed in grams per 100 grams of body weight.

Table S2. Content of basic minerals in rat organs.

Groups/ tissue (μg/g).	Standard	Macrogenistein	Microgenistein	Nanogenistein
**Kidney**				
Ca	102.4 ± 6.7	101.8 ± 5.6	**122.3 ± 16.4**[e]	**19.47 ± 1.04**[a]
Na	1480.7 ± 64.1	1435.6 ± 53.7	1530 ± 9.4	**1587 ± 41.6**[e]
K	2635.9 ± 57.7	2389.3 ± 36.3	2399 ± 21.7	2720.4 ± 44.3
Mg	88.12 ± 6.456	**168.8 ± 3.31**[a]	**172.2 ± 3.22**[a]	**163.7 ± 1,79**[a]
**Brain**				
Ca	15.6 ± 12.2	**84.9 ± 51.8**[f]	**54.8 ± 18.5**[a]	**35.8 ± 21.4**[e]
Na	1238.1 ± 62.4	1262.5 ± 34.7	1264.5 ± 53.5	**1318.6 ± 47.1**[b]
K	3453.8 ± 94.2	3911.9 ± 34.8	**3906.3 ± 112.5**[a]	**4091.8 ± 167.7**[a]
Mg	170.9 ± 28.5	172.6 ± 8.53	166.8 ± 6.93	173.5 ± 9.37
**Heart**				
Ca	9.838 ± 1.32	**64.9 ± 19.1**[a]	**74.83 ± 38.21**[f]	**25.44 ± 10.1**[f]
Na	1237.7 ± 79.84	1210.8 ± 204.8	**1053.2 ± 213.7**[e]	**1130.6 ± 92.4**[e]
K	2698.5 ± 135.4	2723.8 ± 353.5	**2215.7 ± 281.8**[a]	**2455.9 ± 261.8**[e]
Mg	171.8 ± 15.43	**272.9 ± 101.8**[b]	177.5 ± 20.4	**193.4 ± 13.45**[c]
**Liver**				
Ca	9.033 ± 1.14	**64.41 ± 6.63**[a]	**59.72 ± 13.8**[a]	**17.13 ± 5.165**[a]
Na	841.5 ± 143.3	**1607.5 ± 310.5**[a]	**1715.5 ± 419.6**[a]	825.3 ± 171.7
K	3317.9 ± 168.5	3336.6 ± 285.2	**3631 ± 42.39**[a]	3599.5 ± 350.4
Mg	216.9 ± 6.78	221.9 ± 8.185	209.5 ± 17.69	**245.4 ± 26.47**[b]
**Spleen**				
Ca	11.48 ± 5.756	9.777 ± 0.594	10.54 ± 0.836	**26.06 ± 12.20**[d]
Na	806 ± 120.4	744.9 ± 21.9	784.9 ± 11.4	833.9 ± 79.9
K	4245.5 ± 238.3	4201.5 ± 60.9	4131.9 ± 51.8	**4589.6 ± 184.9**[d]
Mg	200.9 ± 10.6	**190 ± 1.299**[b]	**181.1 ± 1.277**[a]	**222.1 ± 12.48**[f]
**Femur** (mg/g)				
Ca	134.41 ± 5,94	134.76 ± 2.71	127.35 ± 8.54	**530.73 ± 50.92**[a]
Na	3.87± 0.35	3.77 ± 0.09	**3.47 ± 0.38**[f]	3.99 ± 0.28
K	1.24 ± 0.15	1.19 ± 0.15	1.20 ± 0.27	**1.55 ± 0.28**[e]
Mg	2.47 ±0.11	2.39 ± 0.05	**2.23 ± 0.18**[a]	**1.94 ± 0.22**[a]

a - $p \leq 0.001$; b - $p \leq 0.02$; c - $p \leq 0.01$; d - $p \leq 0.01$; e - $p \leq 0.05$; f - $p \leq 0.005$ compared to the standard group. Results are presented as means ± SEM.

Table S3. Cancer induction in 7,12-dimethylbenz[a]anthracene treated rats in relation to supplementation [17,18].

supplementation	rat's number	tumor weight (g) (mean±SD) (week 20)	number of tumor per rat (week 20)	the incidence of tumors per rat in time (weeks of rats life)
standard	1	0.90±0.78 (0.1-2.7)	9	17
	2	1.84±3.35 (0.1-7.8)	5	16
	3	0.43±0.30 (0.1-0.8)	4	19
	4	0.45±0.40 (0.1-1.1)	6	18
	5	1.02±1.03 (0.1-2.6)	6	16
	6	0.60±0.00 (0.6)	2	19
	7	1.36±0.92 (0.3-1.96)	3	17
	8	0.61±0.20 (0.47-0.75)	2	17
Macrogenistein	1	2.35±1.96 (0.9-5.14)	4	17
	2	0.52±0.47 (0.14-1.38)	6	17
	3	0.70±0.52 (0.33-1.29)	3	19
	4	1.83±2.48 (0.13-6.39)	6	18
	5	1.74±1.79 (0.24-4.07)	4	17
	6	0.34	1	19
	7	1.58±1.14 (0.29-2.42)	3	17
	8	1.13±1.28 (0.25-3.03)	4	17
Microgenistein	1	0.89±0.40 (0.6-1.17)	2	20
	2	-	-	-
	3	6.11	1	18
	4	1.38±0.69 (0.89-1.86)	2	20
	5	3.31	1	17
	6	2.77±1.81 (0.69-3.89)	3	18
	7	1.45±1.40 (0.19-2.96)	3	18
	8	0.66±0.78 (0.11-1.21)	2	19
Nanogenistein	1	1.01±1.02 (0.1-2.43)	4	19
	2	4.58±2.12 (3.08-6.08)	2	17
	3	0.28±0.20 (0.09-0.48)	3	19
	4	0.20±0.07 (0.1-0.26)	4	20
	5	1.92±3.74 (0.11-8.61)	5	14
	6	1.57±1.94 (0.06-4.41)	4	18
	7	5.03±6.32 (0.56-9.50)	2	18
	8	0.30±0.16 (0.18-0.41)	2	20

Table S4. Histopathological examination of rats tumors [17,18].

Supplementation	Tumor grade	The mean number of mitoses in the field of view area*
Standard	Adenocarcinoma 2 grade	$1.79 \pm 1.25^{a,b,c}$
Macrogenistein	Adenocarcinoma 2 grade	$4.46 \pm 2.38^{a,d}$
Microgenistein	Adenocarcinoma 3 grade	$7.33 \pm 1.57^{b,d}$
Nanogenistein	Adenocarcinoma 3 grade	5.82 ± 1.57^{c}

Data are expressed as mean ± SD (standard deviation). Values sharing letters (a: standard, b: macro genistein, c: micro genistein, d: nano genistein) indicate statistically significant differences between groups ($p<0.01$). *Mitoses were counted in slides from randomly selected tumors in 15 fields of view with a 40× objective magnification.

1. Banys, K.; Giebultowicz, J.; Sobczak, M.; Wyrebiak, R.; Bielecki, W.; Wrzesien, R.; Bobrowska-Korczak, B. Effect of Genistein Supplementation on the Progression of Neoplasms and the Level of the Modified Nucleosides in Rats With Mammary Cancer. *Vivo Athens Greece* **2021**, *35*, 2059–2072. https://doi.org/10.21873/invivo.12475.
2. Banys, K.; Jelińska, M.; Wrzosek, M.; Skrajnowska, D.; Wrzesień, R.; Bielecki, W.; Bobrowska-Korczak, B. Inflammation Factors and Genistein Supplementation in Cancer—Preliminary Research. *Curr. Issues Mol. Biol.* **2024**, *46*, 2166–2180. https://doi.org/10.3390/cimb46030140.
